# Investigating Connectivity Deficits in Alzheimer’s Disease Using a Novel 3D Bioprinted Model Designed to Quantify Neurite Outgrowth

**DOI:** 10.3390/bioengineering12030245

**Published:** 2025-02-28

**Authors:** Chloe Whitehouse, Ellie Bravington, Anirudh Patir, Wei Wei, Janet Brownlees, Yufang He, Nicola Corbett

**Affiliations:** 1 MSD (UK) Limited, 120 Moorgate, London EC2M 6UR, UK; ellie.bravington@msd.com (E.B.);; 2 Merck & Co., Inc., Rahway, NJ 07065, USA; yufang.he@merck.com

**Keywords:** 3D bioprinting, Alzheimer’s disease, iPSC culture, neurite outgrowth

## Abstract

Here, we present a novel 3D bioprinted model of the forebrain cortex designed to quantify neurite outgrowth across a hydrogel bridge. To validate this model, we cultured Alzheimer’s disease (AD) forebrain cortical populations derived from human iPSCs carrying APP (amyloid precursor protein) mutations (K670M/N671L + V717F). Neurite and synapse formation were significantly impaired in 3D AD mutant cultures compared to controls, but this was not replicated in 2D, highlighting deficits in these traditional 2D cell culture models. To investigate the mechanisms underlying impaired neurite outgrowth in 3D and 2D models of AD, we assessed amyloid-β dysfunction, mitochondrial health, and oxidative stress in both conditions. In the 3D model, APP mutant cultures exhibited reduced mitochondrial membrane potential and fragmented networks, indicating dysfunction and potential cellular energy deficits. Additionally, elevated oxidative stress and proteostasis disruption were identified in the 3D AD models as indicators of cellular damage, which may be limiting neurite extension. Furthermore, transcriptomic (bulk RNA-Seq) analysis revealed distinct differences in gene expression pathways between 2D and 3D models of AD, suggesting alternate underlying mechanisms of disease pathology between the culture conditions. This study demonstrates the functionality of this novel 3D bioprinted model for quantifying neurite connectivity and identifying underlying disease mechanisms.

## 1. Introduction

The study of neurological diseases, including Alzheimer’s disease (AD), has been limited by a reliance on two-dimensional (2D) in vitro models, as these models can fail to replicate the complex pathological changes and intricate three-dimensional (3D) architecture of the human brain [1]. AD is often characterised by amyloid-β (Aβ) and tau dysfunction; however, progressive synaptic loss and neurite degeneration, alongside changes to mitochondrial function, oxidative stress, and proteostasis, are also associated with disease progression [2]. These pathological hallmarks ultimately lead to memory deficits and cognitive decline. While 2D cell culture systems have provided valuable insights into AD, they arguably lack the true spatial, mechanical, and biochemical cues that are necessary to fully recapitulate cell–cell interactions and network-level pathologies [3]. Consequently, there is a growing demand for advanced 3D culture systems which can capture the intricacies of neuronal maturation, synaptic connectivity, and pathological cellular stress responses.

Existing 3D neural models, including organoids and neurospheroids, offer considerable advantages over traditional 2D cultures, such as enhanced neuronal differentiation, maturation, and network formation [4,5]. However, these systems often face technical challenges that limit their implementation in quantitative assays. Neural organoids, while physiologically relevant, are time-intensive to generate, lack reproducibility, and are challenging to image due to their dense cellular architecture. Neurospheroids and assembloids, while faster to produce, similarly suffer from imaging limitations which hinder accurate quantification of neurite growth parameters [6].

Alongside traditional 2D culture systems, transgenic rodent models have also been instrumental in studying AD. In vivo models benefit from the ability to track disease progression over time and correlate underlying pathologies with behavioural studies. The broad availability of different transgenic models allows for the investigation of both Aβ and tau pathology within the context of a functional brain, and the interactions of all cell types (including the blood–brain barrier and microglia) alongside the presence of biochemical and biomechanical cues allow a deep understanding of the disease pathology [7]. In contrast, advanced 3D in vitro models offer a controlled and human-relevant microenvironment, and while they may lack whole-organism interactions, these models can excel in their practical advantages, including cost-effectiveness, scalability, and compatibility with high-content assay formats. Additionally, the use of animal models presents ethical concerns, and efforts should be made to refine, reduce, and replace the use of these model systems within all research studies [8], paving the way for a new generation of complex in vitro models.

To address these challenges, we developed a novel tri-matrix 3D bioprinted model of the human forebrain cortex, containing glutamatergic neurons, GABAergic neurons, astrocytes, and microglia, to enable real-time quantification of neurite outgrowth and synaptic connectivity. This platform incorporates a hydrogel bridge within a scaffold-based 3D bioprinted construct, providing a controlled and optically clear area within the model for monitoring the growth of neural projections over time. By optimising the hydrogel bridge stiffness to slow cell migration across matrices, the system facilitates high-resolution imaging while maintaining cell viability and promoting neuronal differentiation. The 96-well plate format of this model also supports medium-throughput applications, enabling robust and reproducible quantification of neurite outgrowth metrics across multiple replicates. This advancement addresses critical limitations in current 3D culture systems, paving the way for its use as a screening platform for therapeutics targeting neurite regeneration and connectivity deficits.

To validate this 3D bioprinted platform, we applied it to model AD using human induced pluripotent stem cell (iPSC)-derived forebrain cortical populations carrying APP (amyloid precursor protein) mutations. The Swedish (K670M/N671L) and Indiana (V717F) mutations in APP promote increased Aβ production, particularly the aggregation-prone isoform of Aβ42, which drives pathology in familial AD [9,10]. Previous studies have shown that Swedish and Indiana APP mutations are able to disrupt neuronal growth, synaptic function, and mitochondrial health in vitro and in vivo [11]. Three-dimensional culture systems have demonstrated the ability to amplify pathological phenotypes across many diseases, likely due to increased complexity in mimicking biomechanical properties of cell–cell interactions and pathological changes to the extracellular matrix [12,13].

In the present study, we demonstrate that 3D APP mutant forebrain cortical populations exhibit fragmented, shorter neurites with reduced branching complexity compared to isogenic controls. Notably, these phenotypes were absent or significantly attenuated in APP mutant cultures grown under 2D conditions, highlighting the importance of the 3D scaffold for capturing these disease-relevant mechanisms. These findings align with clinical observations of synaptic loss and neurite degeneration in AD patients [14]. In addition to the ability to model neurite outgrowth, the tri-matrix 3D bioprinted model provides a platform for other assay formats, including live assays, protein extraction, and cell retrieval from the scaffold. We use these quantitative measurements to investigate underlying cellular pathologies in the AD models which may contribute to the observed neurite outgrowth and connectivity changes. Mitochondrial dysfunction and increased oxidative stress are well-recognised contributors to neurite degeneration, as mitochondria are critical for ATP production, calcium buffering, and energy supply along extending neurites [15]. In this study, we demonstrate that APP mutant neurons in 3D culture exhibit significant mitochondrial dysfunction, increased oxidative stress, and impaired proteostasis, providing mechanistic insights into the observed neurite outgrowth deficits. Importantly, these pathological features were more pronounced in 3D cultures compared to 2D, likely due to the increased energy demands and spatial constraints of the 3D environment.

Taken together, this study establishes a medium-throughput, 3D bioprinted model that enables real-time, high-resolution quantification of neurite outgrowth and connectivity. By applying this platform to develop a 3D model of AD, we demonstrate its ability to recapitulate key pathological features, including impaired neurite growth, mitochondrial dysfunction, and cellular stress, which are not observed in 2D systems. The scalability and compatibility of the model with automated imaging technologies make it a promising tool for drug screening and mechanistic studies of neurodegenerative diseases. Future applications of this platform could extend to other neurological disorders characterised by connectivity deficits, such as Parkinson’s disease, traumatic brain injury, and neurodevelopmental disorders, further underscoring its versatility and translational potential.

## 2. Materials and Methods

### 2.1. iPSC Culture

The human healthy control iPSC line (XCL-1) was CRISPR-edited by Sigma-Aldrich (Gillingham, UK) to include Indiana and Swedish (KM670/671NL + V717F HOMO) APP mutations. Both healthy and APP mutant iPSCs were plated on 10 µg/mL matrigel (Gibco, Swindon, UK) and were maintained in mTESR plus (STEMCell Technologies, Cambridge, UK). The cells were passaged every 5–6 days using ReLeSR (STEMCell Technologies, Cambridge, UK). ROCK inhibitor Y27632 (10 µM) was included in culture media for 24 h after passaging to improve cell survival. Human iPSCs from the same line (XCL-1) were differentiated into induced microglia-like cells (iMGLs) by BrainXell (Madison, WI, USA). All iPSC lines were procured and assessed by internal stem cell approval committees to verify the ethical procurement of cells with appropriate informed patient consent documentation.

### 2.2. iPSC Differentiation into Neural Progenitor Cells

The generation of neural progenitor cells (NPCs) from iPSCs was performed using the SMADi Neural Induction Kit from STEMCell Technologies, following the Monolayer Culture Protocol as per the manufacturer’s protocols. Briefly, iPSCs were dissociated using Gentle Cell Dissociation Reagent at 37 °C for 8–10 min, and cell aggregates were broken up by pipetting the suspension 3–5 times using a 1 mL pipettor. Cells were added to a 15 mL falcon containing DMEM/F12 and were centrifuged for 300× *g* for 5 min. Cells were resuspended at a final concentration of 2 × 10^5^ cells/cm^2^ in Neural Induction Medium with SMADi supplement on 6-well plates coated with Geltrex (Gibco) (1 h at 37 °C). ROCK inhibitor Y27632 (10 µM) was included in culture media for 24 h to improve cell survival. Daily media changes were performed until Day 7, when cells were passaged using Accutase (Gibco) and replated on Geltrex-coated 6-well plates at a concentration of 2 × 10^5^ cells/cm^2^ in Neural Induction Medium with SMADi supplement. This process was repeated when cells were sufficiently expanded for passage on Day 14, and daily media changes were continued until Day 21. At Day 21, NPCs were fully differentiated and were passaged into Geltrex-coated 6-well plates at a concentration of 1.25 × 10^5^ cells/cm^2^ in Neural Progenitor Medium (STEMCell Technologies). After Day 21, the Neural Progenitor Medium was changed 3 times per week. Cells were used for 3D bioprinting or 2D differentiation between Day 28 and Day 35, and NPCs were not used beyond P4. Cells from each batch were evaluated for expression of NPC markers using immunostaining for SOX1 and SOX2 before subsequent differentiation.

### 2.3. Two-Dimensional Differentiation of NPCs into Forebrain Cortical Populations

NPCs were maintained and expanded in Neural Progenitor Medium (STEMCell Technologies) until differentiation. Forebrain Neuron Differentiation and Maturation kits from STEMCell Technologies were used to differentiate NPCs as per the manufacturer’s instructions. Briefly, NPCs were passaged using Accutase as described in the previous section and plated in 96-well plates at a concentration of 0.8 × 10^5^ cells/cm^2^. After 3–5 days, when cells had reached 80% confluence, the media was changed from Neural Progenitor Medium into Forebrain Neuron Differentiation Medium. Daily full media changes of 200 µL Forebrain Neuron Differentiation Medium were performed for 5 days. On Day 6 of forebrain differentiation, the media was changed to 200 µL Forebrain Neuron Maturation medium without passage. Cells were maintained in Forebrain Neuron Maturation medium for a further 15 days with half media changes (100 µL) three times per week. After 15 days in Forebrain Neuron Maturation medium, the media was changed to iMGL maintenance medium: Advanced DMEM/F12 without Phenol Red, 1× B27 supplement (Gibco), 1× N2 supplement (Gibco), 1× non-essential amino acids (NEAA) (Gibco), 1× GlutaMAX (Gibco), 2X ITSG solution (Gibco), 400 µM monothioglycerol (Sigma-Aldrich, Gillingham, UK), 5 µg/mL human insulin (Gibco), 25 ng/mL m-CSF (preprotech, London, UK, and 20 ng/mL IL-34 (preprotech). iMGLs were thawed, and a final concentration of 5% of total cells was added to the forebrain population cultures. Forebrain cultures with iMGLs were maintained for 48 h before assay.

### 2.4. Three-Dimensional Bioprinting of NPCs

Three-dimensional bioprints were created using the RASTRUM™ bioprinter (Inventia Life Science (Sydney, Australia)). A full methodological description of the RASTRUM operation was performed as described in a previous publication [16]. ROCK inhibitor Y27632 (10 µM) was included in NPC culture media 24 h prior to bioprinting to improve cell survival during the print process. The model was designed using the RASTUM™ cloud, with multimatrix conformations “Triple Matrix—Large Plug” with custom spacing used for the large model and the “Triple Matrix—Imaging Model” used for the imaging model. Three hydrogel matrices from Inventia Life Science were trialled for the ability to facilitate NPC expansion and differentiation upon recommendation from Inventia Life Science: Px03.36 (3 kPa stiffness + RGD, IKVAV, YIGSR, with full-length hyaluronic acid protein), Px02.36 (1.1 kPa stiffness + RGD, IKVAV, YIGSR, with full-length hyaluronic acid protein), and Cx02.99161 (1.1 kPa stiffness + RGD, IKVAV, YIGSR, with full-length hyaluronic acid protein and laminin-211). Concentrations of peptides and proteins, alongside other components of hydrogel matrix formulation, are proprietary knowledge of Inventia Life Sciences. To create a model which could facilitate NPC expansion and differentiation in outer compartments, with later neurite growth into the hydrogel bridge, the following matrices were selected: Px03.36 for the hydrogel bridge and Cx02.99161 for the cell-laden matrices. Models were bioprinted into 96-well plates, and plate maps for each experiment were created on the RASTRUM™ cloud software. Wells used for 2D control experiments were pre-coated with Geltrex (1 h at 37 °C), which were washed and replaced with 200 µL Neural Progenitor Medium before commencing the print process. Immediately before bioprinting, bioink fluids were thawed at room temperature, and the printer was greenlighted according to manufacturer instructions. Once thawed, bioink fluids were added to the RASTRUM™ cartridge for inert base bioprinting into the wells of the 96-well plate which would contain 3D structures. During inert base printing, Accutase was added to NPC cultures to dissociate cells into a single-cell suspension. Following dissociation, trypan blue was added, and live cells were counted. A total of 3.2 × 10^6^ NPCs were used per 200 µL of print activator fluid. The total volume of activator fluid is determined by the RASTRUM software following print design. Total NPCs were centrifuged for 300× *g* for 5 min and resuspended in the total required volume of print activator fluid. After inert base printing was complete, the cell-activator suspension was added to the RASTRUM™ cartridge. The RASTRUM™ cartridge and 96-well plate were placed into the RASTRUM™ bioprinter. Three-dimensional cell-laden structures and hydrogel bridges were printed, and the cell suspension was deposited into Geltrex-coated two-dimensional control wells containing Neural Progenitor Medium. After the print process was complete, the hydrogel was checked to ensure gelation and 200 µL Neural Progenitor Medium was added to the remaining 3D wells. Subsequently, 96-well plates were placed into the incubator at 37 °C for 3–5 days to allow NPCs to expand. Neural Progenitor Medium was replaced every 48 h until differentiation.

### 2.5. Differentiation of 3D Bioprinted Models into Forebrain Cortical Populations

**Figure 1:**
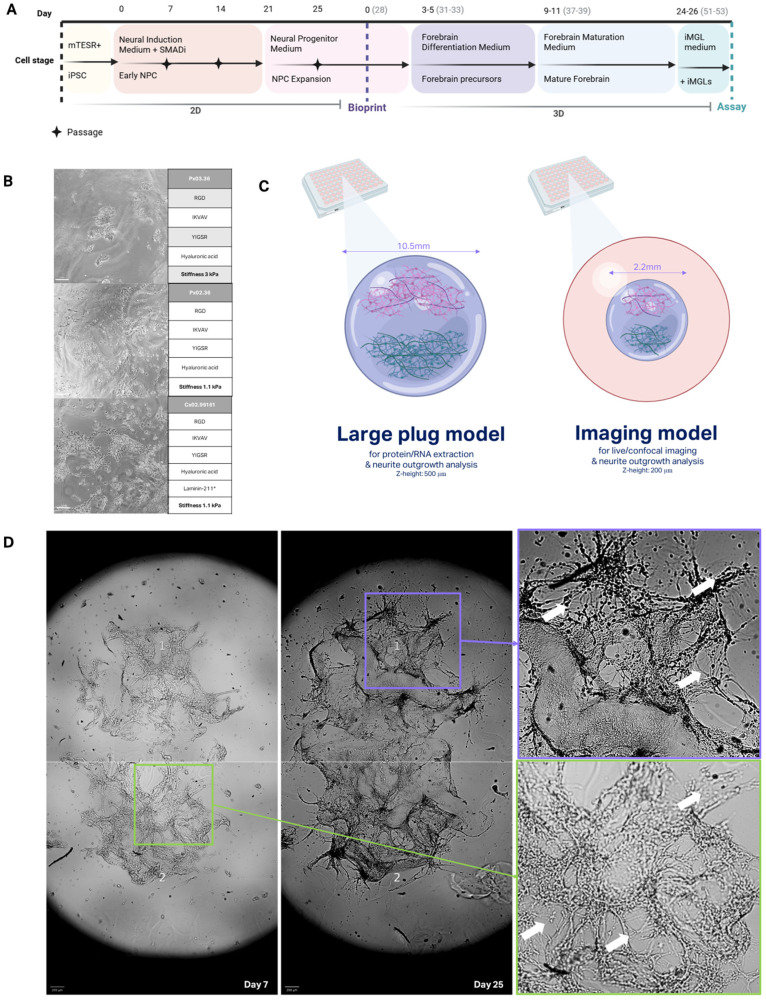
Three-dimensional bioprinted model design and development. (**A**) Culture timeline of 3D model generation from iPSC stage to assay (Day 51–53) (**B**) Brightfield images (10×) of NPCs in matrix selection print run, table corresponding table shows matrix name and key composition. (**C**) Three-dimensional bioprinted model structure and dimensions, empty 96-well is indicated in orange, hydrogel matrix in purple, and cell clusters in green and pink. (**D**) Brightfield images of neuron differentiation and connectivity in 3D imaging model between Day 7 and Day 25, white arrows highlight areas of neurite outgrowth. Scale bars represent 200 µm.

After 3–5 days in Neural Progenitor Medium, cells in 2D wells reached confluence, and NPCs within 3D bioprinted constructs had expanded to form dense pockets of cells within the outer matrix areas. As with 2D differentiation protocols, Forebrain Neuron Differentiation and Maturation kits from STEMCell Technologies were used to differentiate NPCs. Then, 3–5 days post-printing, the media was changed from Neural Progenitor Medium into 200 µL of Forebrain Neuron Differentiation Medium per well. Daily full media changes with Forebrain Neuron Differentiation Medium were performed for 5 days. On Day 6 of forebrain differentiation, media was changed to 200 µL of Forebrain Neuron Maturation medium per well without passage. Cells were maintained in Forebrain Neuron Maturation medium for a further 15 days with half media changes three times per week. After 15 days in Forebrain Neuron Maturation medium, media was changed to 200 µL of iMGL maintenance medium per well (described in the 2D Differentiation Section). iMGLs were thawed, and a final concentration of 5% of total cells was added to the 2D and 3D forebrain population cultures. Forebrain cultures with iMGLs were maintained for 48 h before assay. Live cells were imaged every 8 h for the first 10 days of culture using brightfield 5× magnification on Incucyte S3 (Sartorius, Epsom, UK). For an overview of the culture timeline of 3D model differentiation, see Figure 1A.

### 2.6. Transfection with Neurolight^®^

Cells were transfected with stable expression Neurolight^®^ third-generation HIV-based VSV-G pseudotyped lentivirus (Sartorius) for the neuron-specific Synapsin promoter, which drives expression of red mKate2 in neuronal cell bodies and neurites. Neurolight^®^ was thawed on ice before use. A total of 10 µL per well of Neurolight^®^ Lentivirus was added to a total of 190 µL of Forebrain Differentiation media on Day 1 of forebrain population induction to 3D cultures in 96-well plates. Cells were incubated with the lentivirus for 24 h before a full media change with 200 µL of Forebrain Differentiation medium on Day 2. Cells were imaged on the Incucyte S3 (Sartorius) and on the INcell analyser (Revvity, Wales, UK) at 5× magnification, while cells were live and counterstained with 1× Cell mask green live plasma membrane stain (Thermofisher Scientific, Swindon, UK) following the full culture period.

### 2.7. Cell Viability Assay

Cell viability was indicated by the percent of live cells to total cells from the Live/Dead assay. Live/Dead staining kit (Thermofisher Scientific) was used to assess cell viability. Assays were performed in bioprinted 96-well plates after the full differentiation process was complete. iMGL maintenance media was removed, and the cells were incubated in OptiMEM without phenol red (Gibco) containing 1× NucBlue Live (Thermofisher scientific), 1 mM calcein AM and 2 mM ethidium homodimer I for 30 min. Cells were washed with OptiMEM and imaged on the INcell analyser at 20× magnification. The number of total cells was calculated using NucBlue (nuclei), and live (green) cells and dead (red) cells were counted in each field using Signals Image Artist (SImA) (Revvity). The live/dead cell numbers from five fields and five planes per well were averaged to provide representative results.

### 2.8. Mitochondrial Membrane Potential Assay (TMRM)

Mitochondrial membrane potential was detected with TMRM (tetramethylrhodamine methyl ester) dye. Assays were performed in bioprinted 96-well plates after 48 h of iMGL coculture. iMGL maintenance media was removed, and the cells were incubated in OptiMEM without phenol red (Gibco) containing 1× NucBlue Live (Thermofisher scientific), 1× Cell mask green live plasma membrane stain and 200 µM TMRM (Thermofisher scientific) for 30 min. Cells were washed with OptiMEM and imaged on the INcell analyser at 20× magnification. The region of interest of cells was determined using Cellmask staining, and TMRM intensity per unit area of cell mask staining was calculated for each field using SImA. TMRM intensity was calculated from 5 fields and 5 planes from 12 models per bioprint, for a total of 3 bioprints per condition. These results were averaged to provide representative results.

### 2.9. Reactive Oxygen Species Detection Assay (DHE)

Reactive oxygen species (ROS) production was detected with DHE (Dihydroethidium) dye. Assays were performed in bioprinted 96-well plates after the full differentiation process was complete. iMGL maintenance media was removed, and the cells were incubated in OptiMEM without phenol red (Gibco) containing 1× NucBlue Live (Thermofisher scientific), 1× Cell mask green live plasma membrane stain, and 10 µM DHE (Thermofisher scientific) for 30 min. Cells were washed with OptiMEM and imaged on the INcell analyser at 20× magnification. The region of interest of cells was determined using Cellmask staining, and DHE intensity per unit area of cell mask staining was calculated per each field using SImA. DHE intensity was calculated from 5 fields and 5 planes from 12 models per bioprint, for a total of 3 bioprints per condition. These results were averaged to provide representative results.

### 2.10. Glutathione Detection Assay

Prior to live cell assays (DHE and TMRM), iMGL maintenance media was removed from cells which had been incubated with cocultures for 48 h. Once media was removed from cells, 50 µL of media samples per well were collected in white bottom 96-well plates and used to measure excreted glutathione (GSH). GSH was detected by adding by adding an equal volume of 300 µM monochlorobimane (MCB) and 100 nM Calcein AM in fluorobrite and incubating at 37 °C for 1 h before reading fluorescence on the Clariostar plate reader (BMG Labtech, Aylesbury, UK). Results were calculated from 24 models per bioprint, for a total of 3 bioprints per condition. These results were averaged to provide representative results.

### 2.11. Immunostaining

Immunostaining was conducted in situ in 96-well imaging plates (Phenoplate, Revvity) when cell models had reached maturity. Three-dimensional models and two-dimensional controls were rinsed with PBS before being fixed with 4% paraformaldehyde for 30 min. After rinsing with PBS a further 4 times, cells were permeabilised with 0.1% Triton-X for 20 min. Triton-X solution was rinsed with a PBS wash, and 10% donkey serum (NDS) in PBS was added to block for 3 h at room temperature. Primary antibodies were added in 10% NDS solution and incubated overnight at 4 °C. Three PBS washes were conducted before incubation with secondary antibodies for 2 h at room temperature. Primary and secondary antibodies are detailed in the Supplementary Table S1. Samples were counterstained with DAPI in mountant (Abcam, Cambridge, UK), and cells were subsequently imaged as detailed in the later Microscopy Section.

### 2.12. Microscopy

High-content imaging was performed using an INcell analyser (GE Healthcare, Amersham, UK) with magnifications 5× and 20× for live cell assays and high-content quantification of marker expression following immunofluorescence. Downstream processing of high-content images was conducted using SImA (Revvity) to create analysis protocols, which were performed using the batch analysis feature. Confocal microscopy was conducted on the Nikon SoRa spinning disk confocal microscope at 40× and 63× magnifications. Downstream analysis for confocal images was conducted using Imaris image analysis software (version 10.1.1) (Oxford Instruments, Oxford, UK), including 3D rendering, surface rendering, and neurite tracing.

### 2.13. Neurite Tracing

Neurite tracing was performed from βIII-tubulin immunostaining confocal images. Z-stack images of twenty planes spaced at 1 µm were uploaded into Imaris to create a 3D render of neurons. A filament tracer was used to identify neuronal structures. Firstly, cell somas were identified which were over 8 µm in diameter, and branch points from soma were identified using the filament tracer. Neurite identification was improved using the machine learning feature within the software. All neurites were included in the trace, and the number of neurites per soma was extracted from the image details.

### 2.14. Cell Ratio Analysis

The cell ratio of neurons to astrocytes was calculated from immunostaining images for neuronal marker, βIII-tubulin, and astrocyte marker, S100β. High-content images were taken of 2D and 3D models in 96-well plate formats at 20× magnification using the INcell analyser. Using randomisation, five planes within five fields were selected for the imaging of markers, with a total of twelve wells per condition. SImA 1.4.0 software was used to identify cells using DAPI counter stain and to identify βIII-tubulin+ cells (647 nm) and S100β+ cells (488 nm). The number of S100β+ cells was calculated as a percentage of combined βIII-tubulin+ cells and S100β+ cells.

### 2.15. Quantification of Amyloid-β Isoforms

The Mesoscale Discovery Aβ peptide panel 1 kit was used to quantify the ratios of Aβ present in the cultures. Protein was extracted from 2D and 3D model cultures by removing media, rinsing with cold PBS, and incubating with 50 µL of RIPA buffer with 1× protease inhibitors (both Thermofisher scientific) per well. Protein content from samples was quantified using a BCA assay. Mesoscale Discovery Aβ peptide assay was conducted as per manufacturer instructions. In brief, Mesoscale detection plates were blocked with diluent for 1 h and washed three times before adding 25 µL of detection antibody and 25 µL of samples, controls, and standards (diluted 1:2 and 1:4, three technical replicates per condition) representative results were calculated from 12 models per bioprint, for a total of 3 bioprints per condition. Plates were incubated for a further 2 h before being washed and read on the Mesoscale instrument. A standard curve was used to calculate the concentration of each isoform within the samples. Results were averaged across dilutions and technical replicates and normalised to total protein within the sample.

### 2.16. Bulk RNA-Seq Sample Preparation and Analysis

RNA extraction and bulk RNA-Seq were conducted by Genewiz by Azenta Life Sciences (Oxford, UK). Samples were prepared for bulk RNA-Seq from 24 models per bioprint, for a total of 3 bioprints per condition. Cell pellets were prepared from 2D samples by dissociating mature cultures with Accutase incubation (8–10 min, 37 °C), cells were centrifuged at 300× *g* for 5 min and frozen at −150 °C. Cell pellets were prepared from 3D cultures by incubating with RASTRUM™ fortissimo cell removal solution (Inventia Life Science, Sydney, Australia) for 30 min at 37 °C. Digested hydrogels and cells were removed from wells and centrifuged at 500× *g* for 5 min. Cell pellets were rinsed with PBS to remove remaining hydrogel, and re-centrifuged at 300× *g* for 5 min before freezing at −150 °C.

RNA-Seq library Preparation and Sequencing was performed by GeneWiz. RNA samples were quantified using Qubit 4.0 Fluorometer (Life Technologies, Carlsbad, CA, USA) and RNA integrity was checked with an RNA Kit on Agilent 5300 Fragment Analyzer (Agilent Technologies, Palo Alto, CA, USA). Invitrogen™ ERCC RNA Spike-In Mix (Cat. No.: 4456740) was used following the manufacturer’s instructions. RNA sequencing libraries were prepared using the NEBNext Ultra II RNA Library Prep Kit for Illumina following the manufacturer’s instructions (NEB, Ipswich, MA, USA). Briefly, mRNAs were first enriched with Oligo(dT) beads. Enriched mRNAs were fragmented according to the manufacturer’s instructions. First-strand and second-strand cDNAs were subsequently synthesised. cDNA fragments were end-repaired and adenylated at 3′ends, and universal adapters were ligated to cDNA fragments, followed by index addition and library enrichment by limited-cycle PCR. Sequencing libraries were validated using the NGS Kit on the Agilent 5300 Fragment Analyzer (Agilent Technologies, Palo Alto, CA, USA) and quantified using a Qubit 4.0 Fluorometer (Invitrogen, Carlsbad, CA, USA). The sequencing libraries were multiplexed and loaded on the flow cell on the Illumina NovaSeq Xplus instrument according to the manufacturer’s instructions. The samples were sequenced using a 2 × 150 Pair-End (PE) configuration v1.5. Image analysis and base calling were conducted by the NovaSeq Control Software v1.7 on the NovaSeq instrument. Raw sequence data (.bcl files) generated from Illumina NovaSeq was converted into fastq files and de-multiplexed using Illumina bcl2fastq program version 2.20. One mismatch was allowed for index sequence identification.

The following steps were followed to generate expression matrices from raw sequence reads. We assessed the quality of raw reads (FASTQ format) using FastQC (v 0.12.1) [17]. Subsequently, reads were trimmed for residual adaptor sequences using CutAdapt (v 4.6) [18] and their quality reassessed. Post quality control, reads were aligned to the reference genome (GRCh38 Ensembl 108) [19] using the STAR aligner (v 2.7.11a) [20]. The aligned reads were quantified using featureCounts (Subread package v 2.0.6) to generate a count matrix. A custom script was used to produce a TPM matrix from gene counts and their effective length.

Sample quality control (QC) was conducted using the TPM matrix to examine the effects of batch and outlier samples. We adopted two methods, PCA analysis and sample-to-sample similarity matrices, for this evaluation. PCA analysis was performed on the TPM matrix using the FactoMineR package [21]. The resultant principal components were examined to assess batch effects. The sample-to-sample similarity matrix was generated by calculating pairwise Pearson correlations across all samples based on their TPM-based transcriptomic profiles. The resultant matrix was visualised using a heatmap and clustered using Ward’s hierarchical clustering to examine sample groupings [22].

To identify differentially expressed genes (DEGs), count matrices were used for selected sample groups while accounting for batch effects in the DESeq2 package [15]. Genes were considered significantly up/downregulated if they exhibited an extreme fold change (greater than 1.2 times), a significant adjusted *p*-value (less than 0.05), and minimal normalised expression values across samples (baseMean value greater than 10). Pathway enrichment analysis was conducted using the clusterProfiler package [23] and four databases: GO [16], KEGG [17], MsigDB [18], and Reactome [19]. Only significantly enriched pathways (adjusted *p*-value < 0.05) were considered for functional annotation and interpretation. The results provided insight into the biological processes underlying the expression data. To visualise the overrepresentation of specific pathways, lollipop plots were produced from the top ten enriched terms for each condition and direction.

### 2.17. Quantification and Statistical Analysis

All data are expressed as mean ± SEM unless otherwise stated. Graphs and statistical analysis were made and performed in GraphPad Prism 10.2.2. Statistical tests were always performed using two-way ANOVA with Tukey’s post hoc test unless otherwise indicated. A *p*-value of <0.05 was considered statistically significant. For all graphs displaying statistical analysis, results represent the average of at least 12 models per bioprint across three replicate bioprints, each containing NPCs generated from the same batch of dual SMAD inhibition differentiation.

## 3. Results

### 3.1. Generation of Tri-Matrix 3D Bioprinted Models

Forebrain cortical differentiation protocols were first optimised in 2D before transitioning to 3D systems. Healthy iPSCs were differentiated into NPCs using dual SMAD inhibition, a process that activates neural fate acquisition through inhibition of the BMP and TGF-β pathways. The resulting NPCs expressed hallmark progenitor markers SOX1 and SOX2, demonstrating ectodermal lineage specification (Supplementary Figure S1A). Further differentiation into forebrain cortical neurons produced mixed populations of excitatory and inhibitory neurons alongside astrocytes, which expressed synaptic proteins (Supplementary Figure S1B).

The translation of these differentiation protocols into a 3D platform required careful selection of a hydrogel scaffold that could support NPC proliferation and differentiation. An initial matrix screening was performed to test three candidate hydrogels with varying stiffness and biochemical compositions: Px03.36 (3 kPa, peptides + hyaluronic acid (HA)), Px02.36 (1.1 kPa, peptides + HA), and Cx02.99161 (1.1 kPa, peptides + HA + laminin-211). Limited migration and expansion were observed in Px03.36 and Px02.36. However, the addition of laminin-211 in the Cx02.99161 matrix significantly enhanced cell clustering and neurite outgrowth, highlighting its suitability as a matrix for the forebrain culture (Figure 1D). Laminin is known to play a key role in neuronal adhesion and synapse formation, with the laminin-211 isotype being particularly abundant in the cortex, thus explaining the improved NPC outcomes in Cx02.99161.

Building on these results, a tri-matrix system was developed to allow precise tracking of neurite outgrowth across a hydrogel bridge. In this model, NPCs were bioprinted into two distinct cell populations within matrix Cx02.99161, connected by a bridging hydrogel area made of Px03.36 (3 kPa). This design aimed to delay soma migration while facilitating neurite projection, creating a controlled environment to study neural connectivity (Figure 1C). By Day 7 post-print, cell migration into the bridging zone was restricted, but smaller neurite extensions were populating the area, demonstrating the effectiveness of the matrix in spatially constraining cell soma. By Day 25 post-print, neurite outgrowth successfully bridged the two cell populations, establishing robust connectivity (Figure 1A).

Two distinct tri-matrix model designs were created for specific applications. The “large plug model” spans the width of a 96-well plate and measures 500 µm in Z-height. This model conformation was optimised for downstream analyses such as RNA extraction or biochemical assays (Figure 1C, Large Plug Model). Meanwhile, a smaller imaging model (220 µm width, 200 µm Z-height) was designed for high-resolution confocal imaging, enabling more detailed visualisation of neurite processes and synaptic structures (Figure 1C, Imaging Model). Both designs supported the establishment of neuronal networks, with neurites extending across the hydrogel bridge.

**Figure 2:**
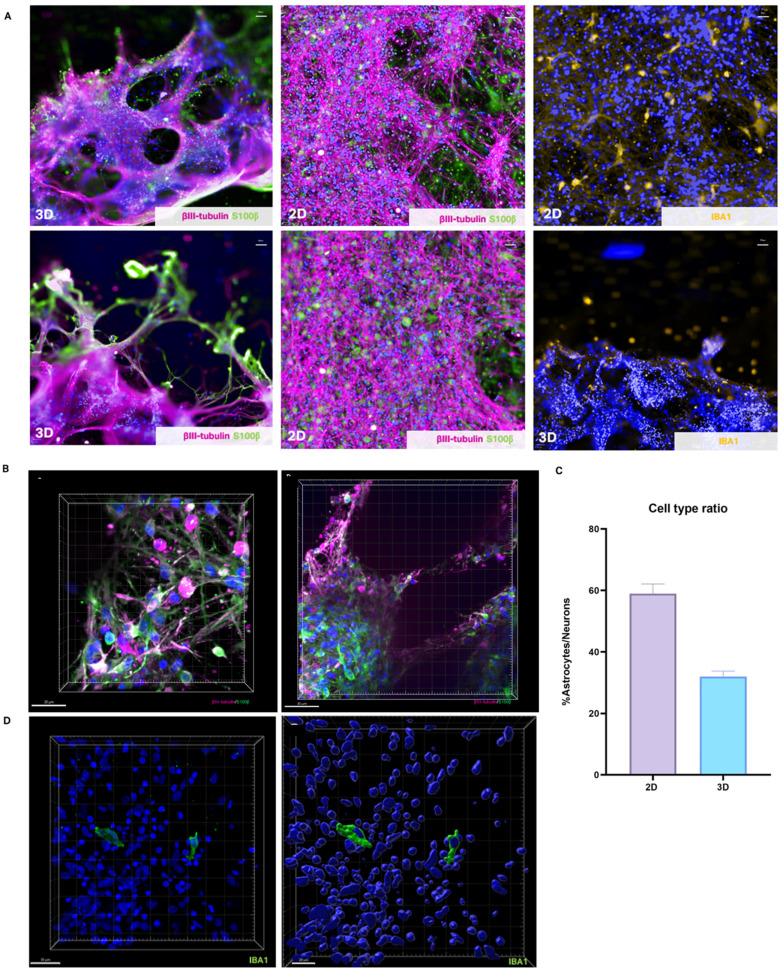
Cell types present in forebrain cortical models. (**A**) Neuronal marker (βIII-tubulin, fuchsia), astrocyte marker (S100β, green), and microglia marker (IBA1, yellow) expression in 2D and 3D culture conditions, with nuclear marker (DAPI, blue). Scale bars represent 100 µm. (**B**) Compiled Z-stack exhibiting morphology and expression of neuronal marker (βIII-tubulin, fuchsia), astrocyte marker (S100β, green) in 3D models, with nuclear marker (DAPI, blue). Scale bars represent 10 µm and 20 µm (left to right). (**C**) Ratio of astrocytic (S100β+) cells to neuronal cells (βIII-tubulin+) in 2D and 3D culture conditions were averaged across 12 models per bioprint, for a total of 3 bioprints per condition (mean ± SEM). (**D**) Compiled Z-stack and surface render of microglial cells (IBA, green) in 3D culture conditions, with nuclear marker (DAPI, blue). Scale bars represent 30 µm and 20 µm (left to right).

**Figure 3:**
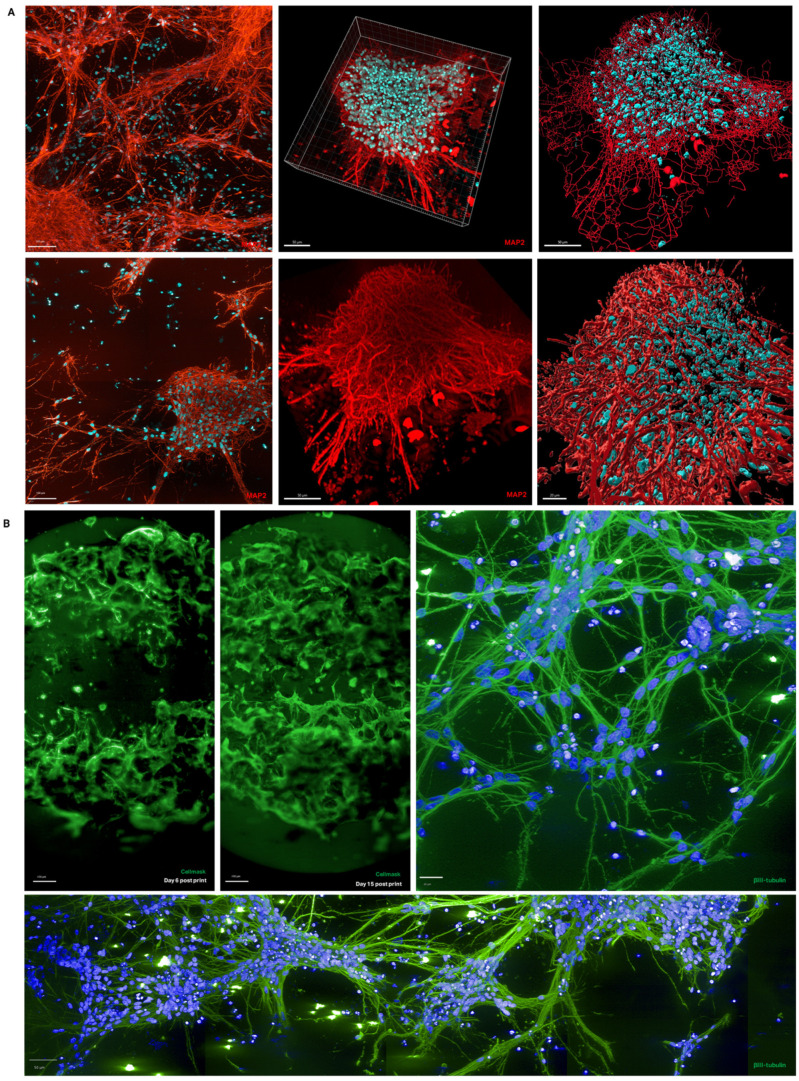
Neural connectivity in 3D model. (**A**) Neuronal marker (MAP2, red) and nuclear marker (DAPI, blue) show neurite extensions within 3D hydrogel matrix. First column shows individual imaging planes, second column shows compiled Z-stack, third column shows filament tracing performed in Imaris software. Scale bars represent 100 µm, 50 µm and 20 µm (left to right). (**B**) Whole well scan of large plug model with live plasma membrane stain (Cell Mask, green) on Day 6 and Day 15 post-bioprinting, and high-magnification images of neurite outgrowth in same model once fixed and stained for neuronal marker (βIII-tubulin, green) and nuclear marker (DAPI, blue). Scale bars represent 400 µm, 400 µm, 20 µm and 50 µm (left to right).

Immunostaining revealed distinct differences in cell composition and morphology between 2D and 3D cultures. Astrocytes, marked by S100β expression, formed monolayers underneath neurons in 2D cultures and comprised approximately 60% of the total population (Figure 2A). In contrast, in 3D cultures, astrocytes were distributed within cell clusters and formed extended projections, representing about 30% of the total population (Figure 2C,D). Induced microglial-like cells (iMGLs) were introduced in the final days of culture, with IBA1 staining confirming their morphology and distribution amongst other cell types within the 3D scaffold (Figure 2B). Mature neurons in 3D cultures expressed the neuronal markers βIII-tubulin and MAP2, and confocal imaging of cells within the hydrogel bridge shows that their neurites are forming networks (Figure 3A,B). Three-dimensional rendering and filament tracing of these confocal images allow neurite networks to be visualised, providing a comprehensive view of neuronal connectivity (Figure 3A).

### 3.2. Neurite Outgrowth and Synapse Formation in 2D and 3D AD Models

**Figure 4:**
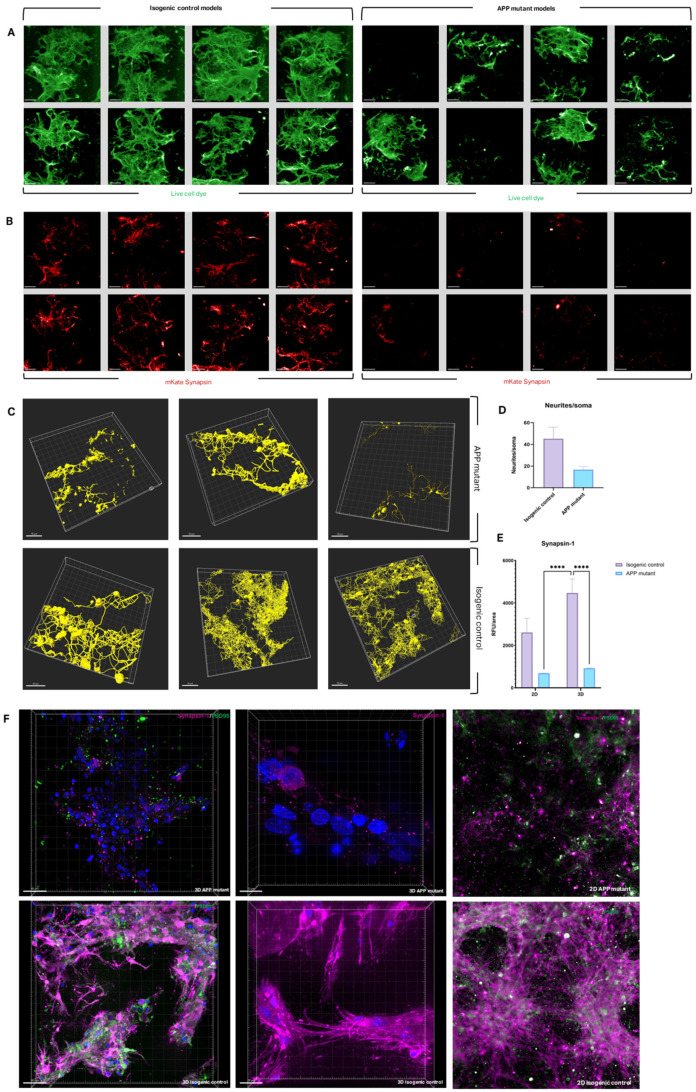
Connectivity and synaptic protein expression in 3D AD models. (**A**) Three-dimensional model morphology in healthy isogenic control models and AD mutant models at Day 25 post-bioprinting using live cell plasma membrane dye (Cell Mask, green). Scale bars represent 400 µm. (**B**) Expression of fluorescent mKate2 synapsin protein (red) in same models as (**A**) following transfection with Neurolight lentivirus. Scale bars represent 400 µm. (**C**) Filament traces of neurite outgrowths (yellow) in hydrogel bridge rendered in Imaris software from βIII-tubulin staining in healthy isogenic controls and AD (APP) mutant models. Each image represents a random field from a model within 1 bioprint. Scale bars represent 50 µm. (**D**) Neurites per soma values qualitatively extracted from confocal images taken from 3 models of 1 bioprint. (**E**) Synapsin-1 expression as quantified from synapsin-1 immunostaining high content imaging; results were averaged across 12 models per bioprint, for a total of 3 bioprints per condition, shown as mean ± SEM with significance indicated as per analysed using 2-way ANOVA. (**F**) Compiled Z-stack images of Synapsin-1 stain (fuchsia) in 3D AD models (APP) and healthy isogenic controls. Scale bars represent 40 µm, 20 µm, and 20 µm (left to right). (**A**–**F**) *p*-value <0.0001 is (****).

The tri-matrix model was next used to study neural network formation in iPSCs carrying APP mutations to model AD in 2D and 3D conditions. These models were also compared to their isogenic controls across assays. Healthy controls demonstrated robust neurite outgrowth across the hydrogel bridge, with neurite bundles connecting cell clusters (Figure 1A and Figure 3B). In contrast, APP mutant cultures exhibited impaired neurite connectivity and reduced cluster formation at the NPC stage, suggesting an early developmental defect (Figure 4A). Synaptic deficits, a hallmark of AD, were also observed in APP mutant cultures. Transfection with mKate-tagged synapsin revealed reduced synapsin expression in APP mutant neurons compared to controls, indicating disrupted synaptogenesis (Figure 4B). Viability assays confirmed significantly lower cell viability in APP mutants, consistent with their impaired connectivity (Supplementary Figure S1C).

Confocal imaging for βIII-tubulin staining was used to render neurite connectivity traces (Figure 4C). Healthy controls exhibited extensive neurite networks, while APP mutants displayed fewer fragmented neurites with visible debris, indicating increased degeneration. Metrics from this filament tracing analysis confirmed a significant reduction in neurites per soma in APP mutants (Figure 4D). Immunostaining for synaptic proteins such as Synapsin-1 and PSD95 revealed significantly reduced expression in APP mutants in 3D cultures, with a less pronounced difference in 2D (Figure 4E,F). These results underscore the enhanced sensitivity of 3D models in detecting subtle synaptic deficits associated with APP mutations.

### 3.3. APP and Amyloid-β Pathology in 2D and 3D AD Models

**Figure 5:**
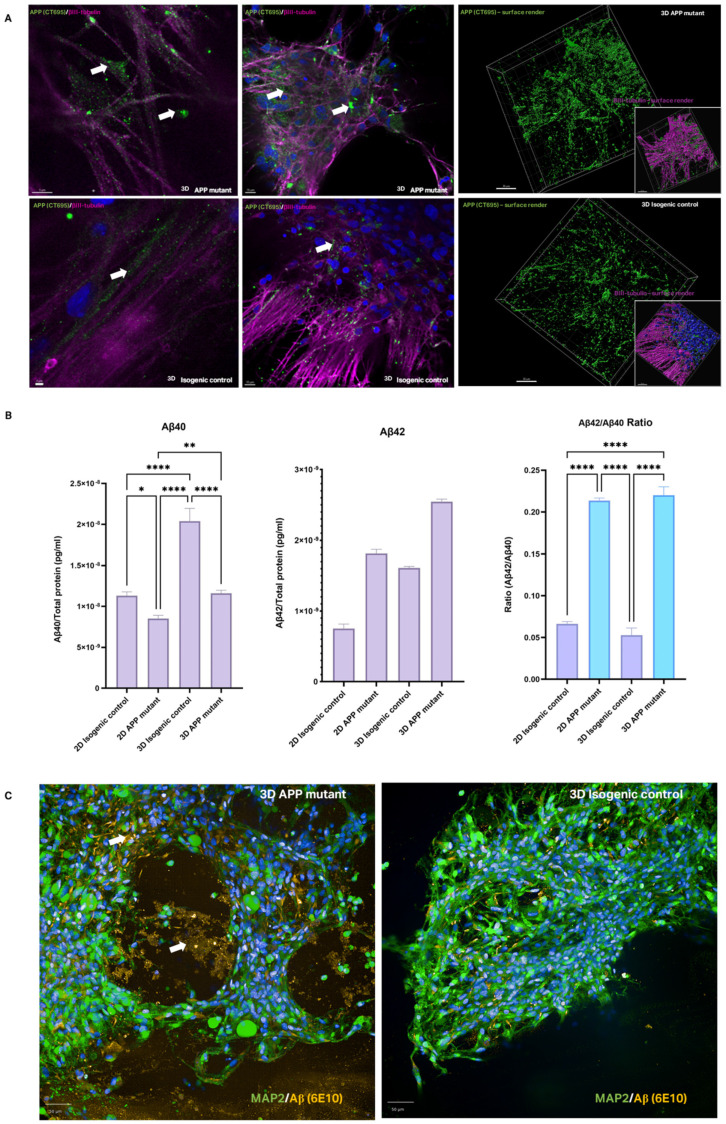
Amyloid-β dysregulation in 3D models. (**A**) Immunostaining of APP C-Terminal fragment (CTF, green) with neuronal marker (βIII-tubulin, fuchsia) and nuclear marker (DAPI, blue) in AD mutants and isogenic controls in 3D. White arrows indicate example areas of APP staining, final column shows surface render of APP staining separate from neuronal stain. Scale bars represent 2 µm, 10 µm, and 30 µm (left to right). (**B**) Aβ isoform quantification for Aβ40 and Aβ42, and the ratio of the two Aβ isoforms in 2D and 3D AD and isogenic control samples. Shown as normalised to total protein in sample and averaged across 12 models per bioprint, for a total of 3 bioprints per condition, statistical significance is indicated as per analysis with 2-way ANOVA (mean ± SEM). (**C**) Immunostaining of amyloid-β protein (6E10, orange) and neuronal marker (βIII-tubulin) with nuclear stain (DAPI, blue) in AD mutant and healthy isogenic control 3D models. White arrows indicate areas of amyloid-β accumulation in hydrogel. Scale bars represent 50 µm. (**A**–**C**) *p*-value < 0.05 is (*), <0.01 is (**), <0.0001 is (****).

The pathological consequences of APP mutations, including altered APP processing and Aβ production, were assessed in both 2D and 3D cultures. APP C-terminal fragments (CTFs) have been implicated in mitochondrial dysfunction and proteostasis deficits [24], and were shown to be more broadly distributed in APP mutants in 3D compared to isogenic controls (Figure 5A). This suggests that the 3D environment may exacerbate proteostasis challenges, highlighting its relevance for studying AD pathology. Aβ levels (Aβ40 and Aβ42) were measured using mesoscale analysis, revealing changes to the Aβ40/42 ratio in 2D and 3D cultures (Figure 5B). Immunostaining also confirmed the localisation of Aβ around cells and within the hydrogel in APP mutant 3D cultures (Figure 5C).

### 3.4. Oxidative Stress and Mitochondrial Dysfunction in 2D and 3D AD Models

**Figure 6:**
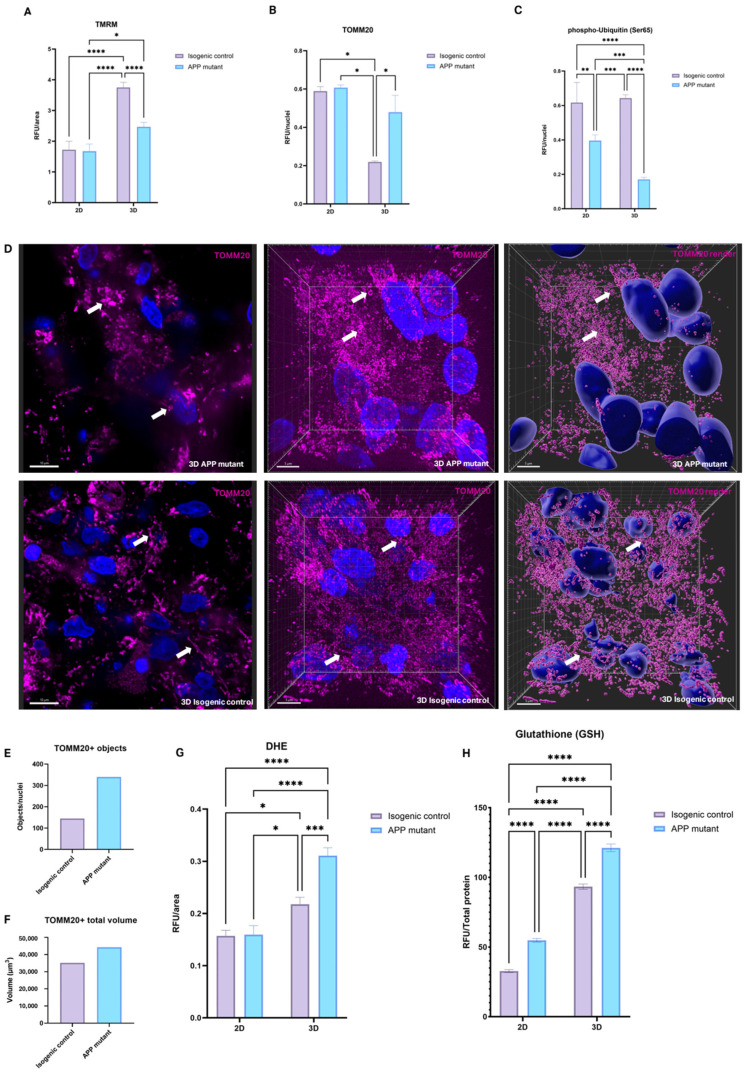
Cellular pathologies in AD 3D models. (**A**) Quantification of live TMRM stain intensity as a measure of mitochondrial health in AD mutant and isogenic control 2D and 3D models, normalised to area of live cells. (**B**) Quantification of TOMM20 immunostaining in AD mutant and isogenic control 2D and 3D models, normalised to nuclei stained with DAPI. (**C**) Quantification of phospho-Ubiquitin immunostaining in AD mutant and isogenic control 2D and 3D models, normalised to nuclei stained with DAPI. For (**A**–**C**), results were averaged across 12 models per bioprint, for a total of 3 bioprints per condition, significance is shown as per analysis with 2-way ANOVA (mean ± SEM). (**D**) Immunostaining of mitochondrial marker (TOMM20, fuchsia) and nuclear stain (DAPI, blue), shown as individual plane (column one), Z-stack (column two), and surface render using Imaris (column three), in AD mutant and isogenic control 3D models (1 model per image within 1 bioprint). White arrows show areas of altered mitochondrial morphology. Scale bars represent 10 µm, 5 µm and 5 µm (left to right). (**E**) Qualitative analysis of image parameter “TOMM20+ objects” from surface render images from 3 models of 1 bioprint (mean ± SEM). (**F**) Qualitative analysis of image parameter “TOMM20+ total volume” from surface render images from 3 models of 1 bioprint (mean ± SEM). (**G**) Quantification of live DHE stain intensity as a measure of ROS production in AD mutant and isogenic control 2D and 3D models, normalised to area of live cells, averaged across 12 models per bioprint, for a total of 3 bioprints per condition (mean ± SEM). (**H**) Quantification of conditioned media GSH assay in AD mutant and isogenic control 2D and 3D models, normalised to total protein of samples and averaged across 24 models per bioprint, for a total of 3 bioprints per condition (mean ± SEM). (**A**–**H**) *p*-value <0.05 is (*), <0.01 is (**), <0.001 is (***), <0.0001 is (****).

Swedish and Indiana APP mutations have been widely suggested to result in mitochondrial dysfunction and increased production of ROS [11]. It has been suggested that poor processing of APP and the resultant changes to amyloid processing pathways cause mitochondrial stress and damage to mtDNA, driving cellular energy imbalances [24]. The dysfunctional mitochondria are not broken down by mitophagy, and this results in oxidative stress. To determine the extent of mitochondrial dysfunction within the models, live cell TMRM assays were used. TMRM is fluorescent when accumulating in mitochondria with an active membrane potential, and thus, an increase in TMRM signal is indicative of increased mitochondrial health. In Figure 6A, there is no significant difference between TMRM fluorescence in isogenic control and APP mutant cultures in 2D. However, in 3D, there is greater fluorescence in the 3D isogenic control compared to the 2D isogenic control. Furthermore, in the 3D cultures, TMRM fluorescence is reduced in APP mutants compared to the isogenic control.

Mitochondrial function was further investigated with immunofluorescent staining for TOMM20, a mitochondrial outer membrane protein, which can give an indication of mitochondrial number. Quantification of TOMM20 staining shows no change between isogenic control and APP mutant cultures in 2D (Figure 6B). However, in 3D cultures, the isogenic control cultures have significantly less TOMM20 expression in comparison to 2D. Furthermore, TOMM20 staining also increases in the APP mutant 3D cultures. An increase in TOMM20 staining could suggest an increased number of mitochondria. However, as TMRM staining is inversely correlated to TOMM20, this indicates that the increased number of mitochondria have a lower membrane potential and therefore are likely to be dysfunctional. Taken together, these results suggest a mitophagy deficit. To investigate this further, cultures were immunostained for phospho-Ubiquitin (pUb). In both 2D and 3D cultures, the APP mutant cells show a decrease in pUb, with the 3D samples showing a more exaggerated change (Figure 6C). This would support the hypothesis of dysfunctional mitophagy in the APP mutant cultures.

To visualise the morphology of the mitochondria in the 3D cultures, TOMM20 was imaged using confocal microscopy. TOMM20 staining shows elongated chains of mitochondria (Figure 6D, indicated by white arrows) in the isogenic control 3D cultures, whereas in the APP mutant 3D cultures, mitochondria appear rounded and fragmented (Figure 6D, indicated by white arrows). TOMM20 and DAPI staining were rendered as surfaces to quantify mitochondrial morphology features, as shown in Figure 6D. The total number of objects and total volume were increased in 3D APP mutants (Figure 6E,F).

An accumulation of dysfunctional mitochondria which are not being cleared by mitophagy can be associated with an increase in oxidative stress. Live DHE (Dihydroethidium) assays were used to investigate the production of ROS within the cultures. DHE is a fluorescent probe for the detection of ROS, specific for superoxide and hydrogen peroxide. As shown in Figure 6G, no difference can be observed between DHE intensity in 2D isogenic control and APP mutant cultures. However, 3D healthy isogenic control samples have higher baseline levels of ROS production. Furthermore, 3D cultures of APP mutant cells have further increased production of ROS.

Glutathione (GSH) is an important cellular antioxidant; thus, the production of GSH from 2D and 3D cultures was measured from media samples. As shown in Figure 6H, 2D cultures have lower levels of secreted GSH than 3D cultures in both isogenic control and APP mutant cells. Additionally, in both culture conditions, APP cells have increased secretion of GSH into the media. In the 3D culture condition, GSH production correlates with increased production of ROS, indicating that antioxidant production may be upregulated in response to increased cell stress.

### 3.5. Transcriptomics of 2D and 3D AD Models

**Figure 7:**
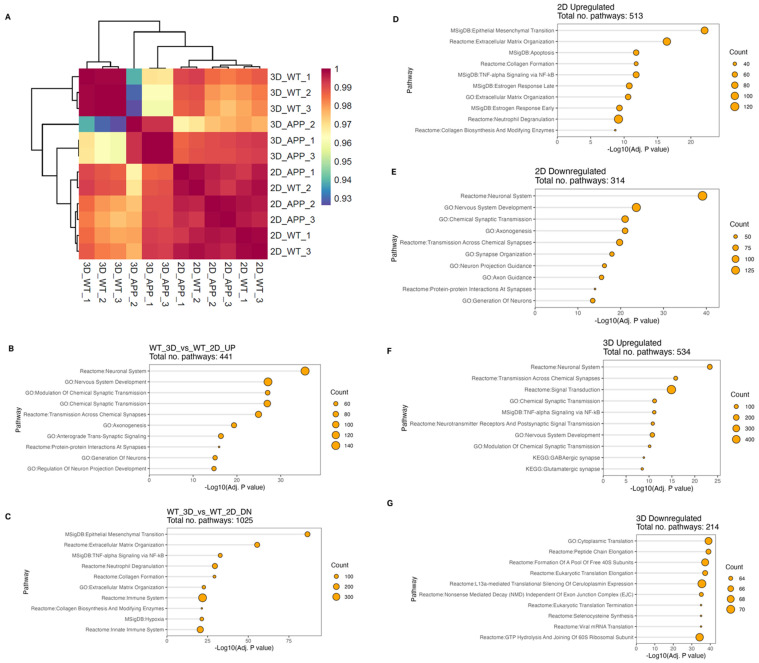
Transcriptomic analysis of AD 2D and 3D models. (**A**) Bulk RNA-Seq analysis of isogenic control and AD mutant 2D and 3D models represented as sample quality control heatmap of Pearson correlation. (**B**–**G**) Over-representation analysis lollipop plots were produced from top ten enriched terms for each condition and direction, where each graph represents. (**B**) Upregulated pathways between isogenic control cells in 3D vs. 2D conditions. (**C**) Downregulated pathways between isogenic control cells in 3D vs. 2D conditions. (**D**) Upregulated pathways between isogenic control cells and AD mutant cells in 2D culture conditions. (**E**) Downregulated pathways between isogenic control cells and AD mutant cells in 2D culture conditions. (**F**) Upregulated pathways between isogenic control cells and AD mutant cells in 3D model conditions. (**G**) Downregulated pathways between isogenic control cells and AD mutant cells in 3D model conditions.

To further investigate the changes between the 2D and 3D cultures of both isogenic and APP mutant cell cultures, cells were removed from within the hydrogel or from the culture plate using enzymatic digestion and pelleted for RNA-Seq from three bioprints per condition. Quality control analysis determining the relationship between samples demonstrates low batch-to-batch variability, with samples within each sample group clustering together regardless of their batch (Supplementary Figure S1E and Figure 7A). Sample groups were compared based on the similarity of their transcriptomic profile (Figure 7A). The heatmap of sample similarity shows that all 2D cultures, including isogenic controls and APP mutants, have a high degree of similarity, thereby grouping together. In contrast, 3D cultures of isogenic control and APP mutant samples group independently, indicative of a lower similarity between these sample groups. In addition, 3D isogenic control cultures show a low degree of similarity to the 2D isogenic control cultures as they group independently.

Differential gene expression (DEG) analysis was conducted to compare (1) 3D vs. 2D isogenic controls, (2) 3D APP mutant vs. 3D isogenic control, and (3) 2D APP mutant and isogenic control. The number of significant upregulated and downregulated DEGs were similar between comparisons ranging from ~ 2800 to 2200 genes (Supplementary Figure S1F). To understand the biology behind these differences, pathway analysis was conducted on DEG for each comparison (Figure 7B–G). On comparing 3D and 2D isogenic controls, upregulated genes in 3D cultures were found in pathways involved in nervous system development, axonogenesis, generation of neurons and synaptic transmission, indicating that 3D cultures are more mature compared to their 2D counterparts. Downregulated genes in 3D cultures compared to 2D cultures of isogenic control cells (Figure 7C) include genes associated with pathways for immune signalling, hypoxia, and extracellular matrix organisation. This indicates that cells in 3D cultures have a less reactive phenotype than in 2D cultures, and that being embedded within hydrogel does not induce hypoxia within the cultures. Downregulation of extracellular matrix reorganisation-associated pathways in 3D cultures could be due to the cells in 3D conditions having retained deposited ECM proteins within the hydrogel, unlike 2D cultures, where ECM proteins will be rinsed off during media changes.

Comparing 2D APP mutants to 2D isogenic control cultures, upregulated genes (Figure 7D) are associated with pathways for immune signalling, cytokine activity, apoptosis, and collagen synthesis. Meanwhile, downregulated genes (Figure 7E) are associated with pathways for nervous system development, synaptic transmission, and axonogenesis. This demonstrates that in 2D cultures, APP mutations are disrupting neuronal growth and synaptic transmission, and making cells take on a more reactive phenotype. Conversely, when comparing 3D APP mutants with 3D isogenic control cultures, upregulated genes (Figure 7F) are significantly enriched in pathways for signal transduction, synaptic transmission, and neurotransmitters. This is at odds with protein analysis work, which demonstrated that synaptic proteins are significantly less expressed in the APP mutant cultures and that neuronal outgrowth was significantly reduced. However, the downregulated genes in 3D APP mutant vs. 3D isogenic control cultures (Figure 7G) are associated with pathways for translation of proteins and ribosomal function. This could suggest that although genes are upregulated, they are not being translated into functional proteins.

In summary, it is evident that there are significantly different changes to cellular mechanisms and protein expression between 2D and 3D cultures due to the culture conditions of the cells (2D vs. 3D), and this is further exaggerated by the disparate changes between the APP mutants in 2D and 3D culture conditions.

Overall, these findings highlight the advantages of using 3D culture systems for modelling neurodevelopmental and neurodegenerative diseases. The tri-matrix model not only recapitulates key aspects of neural connectivity and synaptic organisation but also amplifies disease-relevant phenotypes, providing a powerful platform for studying AD and other disorders.

## 4. Discussion

In this study, we developed a tri-matrix 3D bioprinted scaffold-based model to assess neurite outgrowth and neural connectivity using human iPSC-derived NPCs differentiated into forebrain cortical cocultures. The model was validated in the context of AD using APP mutant NPCs, recapitulating critical features of neurodegenerative pathology. Our findings demonstrate that the tri-matrix 3D system provides a physiologically relevant environment for studying neural interactions and connectivity while addressing the limitations of conventional 2D cultures. The APP mutant model revealed significant reductions in neurite outgrowth, synaptic protein expression, and matrix remodelling, alongside underlying mitochondrial dysfunction, oxidative stress, and proteostasis disruption. Importantly, many of these pathological features were not observed robustly in the corresponding 2D cultures, emphasising the necessity of 3D systems to capture disease-relevant phenotypes.

Considerable progress has been made in the development of neural 3D models, but few systems can be implemented in the quantitative assessment of neurite outgrowth and connectivity [16]. Our tri-matrix 3D bioprinted model addresses two major limitations in this field: optical clarity for high-resolution real-time imaging and compatibility with medium-throughput analysis in 96-well plate formats.

Traditional models, such as neural organoids and neurospheroids, offer insights into brain-like structures but present practical challenges for application. Neural organoids are limited by their labour-intensive setup process and low throughput format, while neurospheroids and assembloids, although generally more scalable, often have dense, intertwined neuronal structures that obscure individual neurite resolution [4,5]. The hydrogel bridge in the tri-matrix model, designed with increased stiffness (3 kPa) to restrict soma migration while permitting neurite extension, creates an optically clear environment where neurite projections can be easily imaged and quantified.

The increased hydrogel stiffness in the central matrix, achieved through higher crosslinking density, reduces pore size and initially prevents large cell soma from populating the hydrogel bridge while still allowing neurite penetration. This ensures that the central region remains sparsely populated, enabling high-resolution imaging and quantitative analyses of neurite growth metrics, such as neurite length, branching complexity, and neurites per soma. Additionally, the total printing time of 32 min per 96-well plate improves model practicality for medium-throughput applications, facilitating its potential use in industrial applications.

While the 28-day culture duration may pose a challenge for high-throughput applications compared to simple 2D assays, this culture time remains significantly shorter than standard organoid protocols, which often require around 100 days for full maturation, while offering the same benefit of a self-assembling complex model system. Additionally, within the 28-day period, transcriptomic analysis reveals that the 3D system achieves a higher degree of neuronal maturation and synaptic connectivity within the 28 days, indicating that this model provides a more translatable model within a shorter time frame.

To demonstrate the translatability and application of the system in disease modelling, we adapted the tri-matrix model to create a model of AD using APP mutant NPCs. In 3D culture conditions, neurite outgrowth across the hydrogel bridge was significantly reduced in APP mutant cells compared to isogenic controls. APP mutant neurites were fragmented, shorter, and exhibited reduced branching complexity, alongside a marked reduction in synapsin-1 expression, indicating impaired synapse formation. In contrast, APP mutant cells cultured under 2D conditions displayed little difference in morphology or synaptic protein expression compared to controls. These findings highlight the ability of the 3D bioprinted system to capture early disease phenotypes that remain undetected in traditional 2D cultures.

Transcriptomic analysis demonstrated greater neuronal maturation in 3D cultures compared to 2D cultures in isogenic controls. Genes related to axonogenesis, synaptic transmission, and nervous system development were upregulated, indicating that 3D environments promote enhanced neuronal differentiation and maturation. However, transcriptomic analysis also showed distinct molecular mechanisms implicated in neurite deficits between the 2D and 3D APP mutant cultures. In 2D conditions, downregulation of genes associated with neuronal growth and synaptic transmission was observed. Conversely, in 3D APP mutant cultures, pathways related to protein translation and ribosomal function were downregulated, consistent with impaired protein synthesis. The downregulation and reduced expression of ribosomal proteins and translation machinery have been previously described in APP models [25,26,27]. Additionally, the relationship between APP and synaptogenesis is complex, with evidence for APP mutations resulting in a synaptogenic effect in some culture conditions [28]. These results suggest that the reduced expression of ribosomal proteins could be seen to result in failure to translate the increased synapse-related genes into functional proteins, which would result in the observed deficits in neurite connectivity and synapse formation.

To understand the cellular mechanisms contributing to neurite outgrowth changes between 2D and 3D APP mutant cultures, we examined mitochondrial function, oxidative stress, and proteostasis, which are well-established hallmarks of AD. APP mutant cultures displayed disrupted Aβ processing, evidenced by altered Aβ40/42 ratios and increased APP C-terminal fragment and Aβ accumulation in the hydrogel. However, significant mitochondrial dysfunction and oxidative stress were only observed in 3D cultures.

Mitochondria play a central role in neurite outgrowth by supplying ATP and calcium buffering to support cytoskeletal remodelling and local energy demands [15]. In 3D APP mutant cultures, we observed reduced mitochondrial membrane potential and fragmented mitochondrial networks. Notably, baseline mitochondrial activity in isogenic 3D cultures was also higher than in 2D cultures, consistent with previous reports of increased energy demands in 3D environments [29,30]. This elevated baseline energy requirement may exacerbate mitochondrial dysfunction in disease contexts, limiting the availability of ATP for neurite extension and maintenance.

Oxidative stress also contributes to cellular damage and mitochondrial dysfunction [2]. APP mutant 3D cultures exhibited elevated ROS levels compared to controls, alongside increased GSH production, indicating a compensatory antioxidant response. Despite this, the production of ROS persisted, likely creating a feedforward cycle of mitochondrial damage, energy deficits, and impaired neurite outgrowth.

Additionally, APP mutant 3D cultures displayed impaired proteostasis, as evidenced by reduced pUb levels. The accumulation of misfolded proteins, including Aβ, further increases cellular stress and compromises mitochondrial function. Together, the combination of mitochondrial dysfunction, production of ROS, and proteostasis disruption in APP mutant 3D cultures provides a mechanistic explanation for the pronounced neurite deficits observed in this system.

While our 3D bioprinted model represents a significant advancement, certain limitations must be addressed. First, the APP mutations used in this study (Swedish and Indiana) primarily affect Aβ processing, and additional models incorporating tau pathology are needed to fully capture the spectrum of AD pathophysiology. The next steps to develop this model further could also include a transition to patient-derived cells to fully capture pathology in sporadic AD patients. Additionally, while bulk RNA sequencing provides valuable insights into gene expression changes, single-cell RNA sequencing could offer a more detailed understanding of cellular heterogeneity within 3D cultures. Additionally, future experiments with this model should incorporate pharmacokinetic analysis to assess drug distribution, metabolism, and clearance within the 3D system, offering critical insights into compound bioavailability and efficacy to provide a robust view of translatability. Finally, the scalability of the 3D bioprinted model for true high-throughput screening applications of thousands of compounds remains to be further optimised, although the 96-well plate format used in this study represents a promising step toward medium-throughput applications.

Although this model system demonstrates clear potential to recapitulate pathologies associated with AD, the field of advanced translational in vitro models remains in its infancy, and in this light, in vivo models of Alzheimer’s disease remain invaluable. In vivo models of AD allow for the study of systemic interactions, neuroimmune responses, and behavioural phenotypes that cannot yet be replicated in vitro [7]. These models provide essential insights into the progression of AD within a living organism, also allowing for the study of blood–brain barrier dynamics, neurovascular interactions, and long-term pathological changes over time. Advanced 3D models, such as this tri-matrix bioprinted model, offer a complementary approach by enabling tightly controlled studies of human-specific neuronal interactions, synaptic dysfunction, and disease phenotypes at the cellular level. By integrating 3D models alongside in vivo studies, it is possible for researchers to refine the experimental design, reducing the number of animals required while improving mechanistic understanding before proceeding to whole-organism studies [31].

## 5. Conclusions

Our findings highlight the limitations of conventional 2D models for studying AD and underscore the value of 3D bioprinted models in capturing the complexity of disease pathology. The 3D environment not only promotes neuronal maturation but also amplifies key features of AD, including synaptic dysfunction, mitochondrial impairment, and oxidative stress. This makes 3D bioprinted models a powerful tool for studying the cellular mechanisms underlying AD and for screening potential therapeutic interventions. The ability to track neurite outgrowth and connectivity in real-time using the hydrogel bridge within this triple-matrix model provides a unique advantage and potential assay output for future studies of neurodegeneration, and serves as a template model for other neurological diseases.

## Data Availability

The datasets presented in this article are not readily available because internal data belong to MSD (UK) Limited, London, UK. Requests to access the datasets should be directed to the corresponding author.

## Supplementary Materials

The following supporting information can be downloaded at: https://www.mdpi.com/article/10.3390/bioengineering12030245/s1.

## References

[1] Whitehouse C., Brownlees J., Corbett N. (2023). 3D models of neurodegeneration: Implementation in drug discovery. Trends Pharmacol. Sci..

[2] Misrani A., Tabassum S., Yang L. (2021). Mitochondrial Dysfunction and Oxidative Stress in Alzheimer’s Disease. Front. Aging Neurosci..

[3] Pereira I., Lopez-Martinez M.J., Samitier J. (2023). Advances in current in vitro models on neurodegenerative diseases. Front. Bioeng. Biotechnol..

[4] Giandomenico S.L., Sutcliffe M., Lancaster M.A. (2021). Generation and long-term culture of advanced cerebral organoids for studying later stages of neural development. Nat. Protoc..

[5] Woodruff G., Phillips N., Carromeu C., Guicherit O., White A., Johnson M., Zanella F., Anson B., Lovenberg T., Bonaventure P. (2021). Screening for modulators of neural network activity in 3D human iPSC-derived cortical spheroids. PLoS ONE.

[6] Brémond Martin C., Simon Chane C., Clouchoux C., Histace A. (2021). Recent Trends and Perspectives in Cerebral Organoids Imaging and Analysis. Front. Neurosci..

[7] Sanchez-Varo R., Mejias-Ortega M., Fernandez-Valenzuela J.J., Nuñez-Diaz C., Caceres-Palomo L., Vegas-Gomez L., Sanchez-Mejias E., Trujillo-Estrada L., Garcia-Leon J.A., Moreno-Gonzalez I. (2022). Transgenic Mouse Models of Alzheimer’s Disease: An Integrative Analysis. Int. J. Mol. Sci..

[8] Grimm H., Biller-Andorno N., Buch T., Dahlhoff M., Davies G., Cederroth C.R., Maissen O., Lukas W., Passini E., Törnqvist E. (2023). Advancing the 3Rs: Innovation, implementation, ethics and society. Front. Vet. Sci..

[9] Lloyd G., Trejo-Lopez J., Xia Y., McFarland K., Lincoln S., Ertekin-Taner N., Giasson B., Yachnis A., Prokop S. (2020). Prominent amyloid plaque pathology and cerebral amyloid angiopathy in APP V717I (London) carrier—Phenotypic variability in autosomal dominant Alzheimer’s disease. Acta Neuropathol. Commun..

[10] Choe M.S., Yeo H.C., Kim J.S. (2024). Simple modeling of familial Alzheimer’s disease using human pluripotent stem cell-derived cerebral organoid technology. Stem Cell Res. Ther..

[11] Fang E.F., Hou Y., Palikaras K. (2019). Mitophagy inhibits amyloid-β and tau pathology and reverses cognitive deficits in models of Alzheimer’s disease. Nat. Neurosci..

[12] Cenini G., Hebisch M., Iefremova V., Flitsch L., Breitkreuz Y., Tanzi R., Doo Y.K., Peitz M., Brüstle O. (2021). Dissecting Alzheimer’s disease pathogenesis in human 2D and 3D models. Mol. Cell. Neurosci..

[13] Sethi M.K., Zaia J. (2017). Extracellular matrix proteomics in schizophrenia and Alzheimer’s disease. Anal. Bioanal. Chem..

[14] Scheff S.W., Price D.A., Schmitt F.A., DeKosky S.T., Mufson E.J. (2007). Synaptic alterations in CA1 in mild Alzheimer disease and mild cognitive impairment. Neurology.

[15] Fame R., Lehtinen M. (2021). Mitochondria in Early Forebrain Development: From Neurulation to Mid-Corticogenesis. Front. Cell Dev. Biol..

[16] Whitehouse C., He Y., Brownlees J., Corbett N. (2023). Three-Dimensional Bioprinting of Human iPSC-Derived Neuron-Astrocyte Cocultures for Screening Applications. J. Vis. Exp..

[17] Simon A. FastQC: A Quality Control Tool for High Throughput Sequence Data. https://www.bioinformatics.babraham.ac.uk/projects/fastqc/.

[18] Martin M. (2011). Cutadapt removes adapter sequences from high-throughput sequencing reads. EMBnet. J..

[19] Zheng-Bradley X., Streeter I., Fairley S., Richardson D., Clarke L., Flicek P. (2017). Alignment of 1000 Genomes Project reads to reference assembly GRCh38. GigaScience.

[20] Dobin A., Davis C.A., Schlesinger F., Drenkow J., Zaleski C., Jha S., Batut P., Chaisson M., Gingeras T.R. (2013). STAR: Ultrafast universal RNA-seq aligner. Bioinformatics.

[21] Lê S., Josse J., Husson F. (2008). FactoMineR: An R Package for Multivariate Analysis. J. Stat. Softw..

[22] Murtagh F., Legendre P. (2011). Ward’s Hierarchical Clustering Method: Clustering Criterion and Agglomerative Algorithm. J. Classif..

[23] Xu S., Hu E., Cai Y., Xie Z., Luo X., Zhan L., Tang W., Wang Q., Liu B., Wang R. (2024). Using clusterProfiler to characterize multiomics data. Nat. Protoc..

[24] Lee S., Kwon D., Shin N., Kong D., Kim N., Kim H., Choi S., Kang K. (2022). Accumulation of APP-CTF induces mitophagy dysfunction in the iNSCs model of Alzheimer’s disease. Cell Death Discov..

[25] Maina M.B., Bailey L.J., Doherty A.J., Serpell L.C. (2018). The Involvement of Aβ42 and Tau in Nucleolar and Protein Synthesis Machinery Dysfunction. Front. Cell Neurosci..

[26] Elder M.K., Erdjument-Bromage H., Oliveira M.M., Mamcarz M., Neubert T.A., Klann E. (2021). Age-dependent shift in the de novo proteome accompanies pathogenesis in an Alzheimer’s disease mouse model. Commun. Biol..

[27] Feng L., Wang G., Song Q., Feng X., Su J., Ji G., Li M. (2024). Proteomics revealed an association between ribosome-associated proteins and amyloid beta deposition in Alzheimer’s disease. Metab. Brain Dis..

[28] Zhou B., Lu J.G., Siddu A., Wernig M., Sudhof T. (2022). Synaptogenic effect of APP-Swedish mutation in familial Alzheimer’s disease. Sci. Transl. Med..

[29] Tidwell T.R., Røsland G.V., Tronstad K.J., Søreide K., Hagland H.R. (2022). Metabolic flux analysis of 3D spheroids reveals significant differences in glucose metabolism from matched 2D cultures of colorectal cancer and pancreatic ductal adenocarcinoma cell lines. Cancer Metab..

[30] Rybkowska P., Radoszkiewicz K., Kawalec M., Dymkowska D., Zabłocka B., Zabłocki K., Sarnowska A. (2023). The Metabolic Changes between Monolayer (2D) and Three-Dimensional (3D) Culture Conditions in Human Mesenchymal Stem/Stromal Cells Derived from Adipose Tissue. Cells.

[31] Sinha S., Wal P., Goudanavar P., Divya S., Kimothi V., Jyothi D., Sharma M.C., Wal A. (2024). Research on Alzheimer’s Disease (AD) Involving the Use of In vivo and In vitro Models and Mechanisms. Cent. Nerv. Syst. Agents Med. Chem..

