# Supplementary material for: Investigating Connectivity Deficits in Alzheimer’s Disease Using a Novel 3D Bioprinted Model Designed to Quantify Neurite Outgrowth
Source: Bioengineering (Basel). 2025 Feb 28;12(3):245. doi: 10.3390/bioengineering12030245 (PMC11939190; doi:10.3390/bioengineering12030245)
Supplement: Supplementary file 1 [file bioengineering-12-00245-s001.zip › Table S1.pdf]

Supplementary Table S1: Primary & secondary antibodies used for immunostaining.

Antibody	Concentration	Cat no., Manufacturer
Donkey anti-mouse Alexa fluor 488	1:1000	A-2120, Thermofisher
Donkey anti-mouse Alexa fluor 568	1:1000	A-10037, Thermofisher
Donkey anti-rabbit Alexa fluor 488	1:1000	A-21206, Thermofisher
Donkey anti-rabbit Alexa fluor 568	1:1000	A-10042, Thermofisher
Donkey anti-rabbit Alexa fluor 647	1:1000	A-31573, Thermofisher
Goat anti-human SOX1	1:50	A24354, Thermofisher
Mouse anti-human Amyloid beta 6E10	1:1000	MA5-51794, Thermofisher
Mouse anti-human BIII-tubulin, NL647 conjugated	1:10	R&D Systems
Mouse anti-human GABBR1	1:500	ab55051, abcam
Mouse anti-human IBA1	1:500	ab5076, abcam
Mouse anti-human MAP2	1:500	M9942, Sigma Aldrich
Mouse anti-human PSD-95	1:500	ab13552, abcam
Rabbit anti-human APP CTF695	1:500	51-2700, Thermofisher
Rabbit anti-human BIII-tubulin	1:500	ab18207, Abcam
Rabbit anti-human NMDAR	1:500	ab109182, abcam
Rabbit anti-human pUbiquitin (Ser65)	1:1000	62802, Cell signalling
Rabbit anti-human S100B	1:100	ab52642, abcam
Rabbit anti-human SOX2	1:50	A24354, Thermofisher
Rabbit anti-human Synapsin-1	1:500	ab18258, abcam
Rabbit anti-human TOMM20	1:1000	ab186735, abcam
Rabbit anti-human vGlut1	1:500	ab227805, abcam
