# Supplementary figures and images for: Investigating Connectivity Deficits in Alzheimer’s Disease Using a Novel 3D Bioprinted Model Designed to Quantify Neurite Outgrowth
Source: Bioengineering (Basel). 2025 Feb 28;12(3):245. doi: 10.3390/bioengineering12030245 (PMC11939190; doi:10.3390/bioengineering12030245)

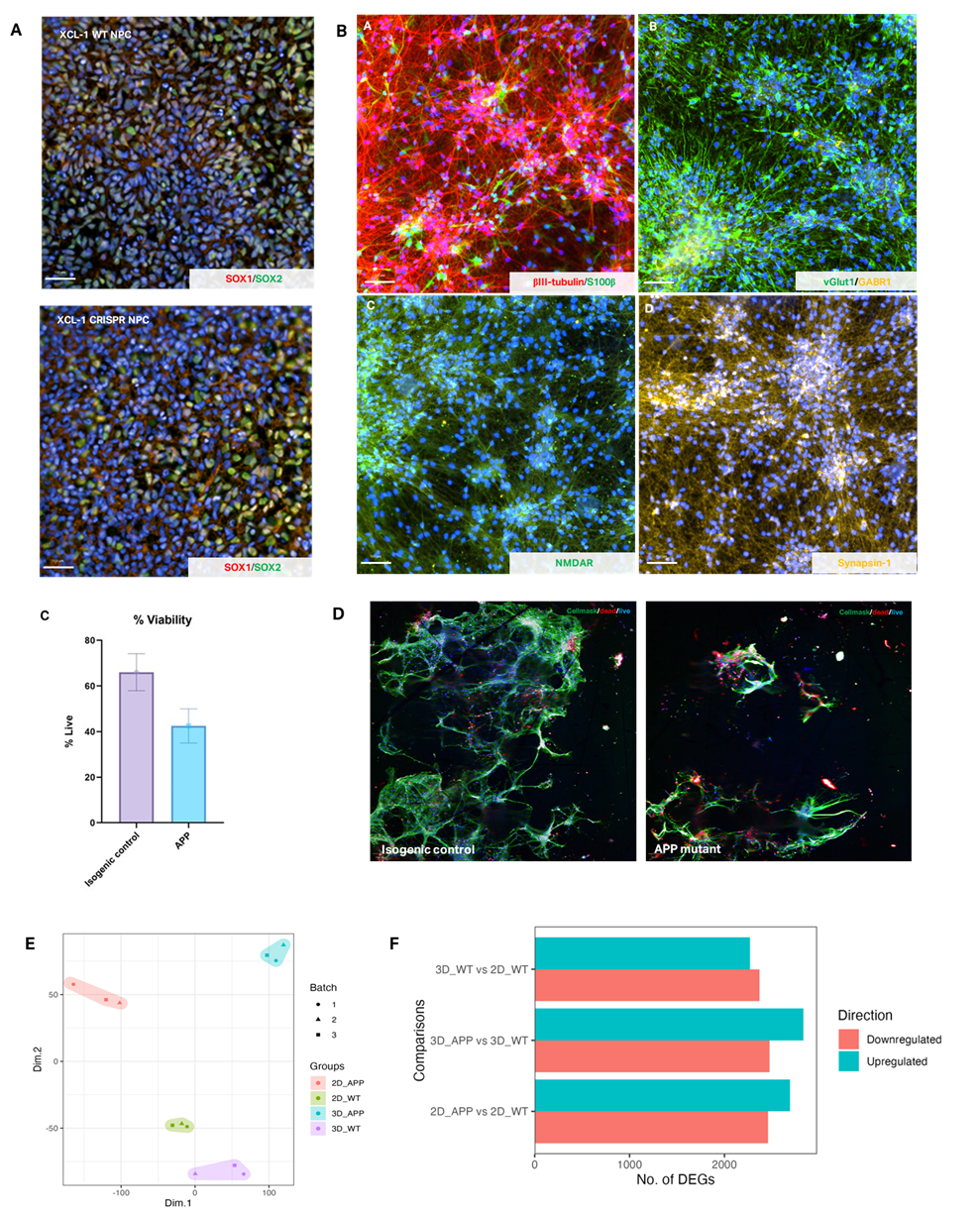

Supplement: Supplementary file 1 [file bioengineering-12-00245-s001.zip › Supplementary Figure S1.bmp]
